# Supplementary material for: Polo-like kinase acts as a molecular timer that safeguards the asymmetric fate of spindle microtubule-organizing centers
Source: eLife. 2020 Nov 2;9:e61488. doi: 10.7554/eLife.61488 (PMC7669271; doi:10.7554/eLife.61488)
Supplement: Supplementary file 1. [file elife-61488-supp1.docx]

**Table S1: Strains**

| Strain | Relevant genotype |
| --- | --- |
| F496 | MATa (wild type from the W303 background) |
| F2071 | MATa, *SPC42-RFP::KanMX6*, *SPC110-GFP::klTRP* |
| F2111 | MATa, *SPC110-dsRed::KanMX6* |
| F2276 | MATa, *SPC42-GFP::HIS3, SPC110-dsRed::KanMx* |
| F2351 | MATa, *SPC72-mCherry::KanMX6, KAR9-GFP::HisMX6* |
| F2741 | MATa, *SPC42-RFP::KanMX6*, *SPC110-GFP::klTRP, cdc5-L158G::KanMX6* |
| F2845 | MATa, *SPC72-mCherry::KanMX6, KAR9-GFP::HisMX6, cdc5-L158G::KanMX6* |
| F2865 | MATa, *SPC42-GFP::HIS3, SPC110-dsRed::KanMx, cdc5-L158G::KanMX6* |
| F3053 | MATa, *ura3-1::ADH1-OsTIR1-9Myc(URA3), cdc5-L158G-yEGFP::HIS3, CDC20-AID::kanMX, SPC72-mCherry::KanMX6* |
| F3172 | MATa, *KAR9-sfGFP(cp8)::KanMX4* |
| F3420 | MATa, *cdc5-L158G-VN::His3MX6, SPC72-VC::KanMX, SPC42-mCherry::KanMX6* |
| F3696 | MATa, *SPC72-GFP::HIS3* |
| F3699 | MATa, *SPC72-GFP::HIS3, cdc5-L158G* |
| F3702 | MATa, *SPC72-GFP::HIS3, SPC110-dsRed::KanMx* |
| F3703 | MATa, *SPC110-dsRed::KanMX6, cdc5-L158G::KanMX6* |
| F3705 | MATa, *SPC110-dsRed::KanMx, SPC72-GFP::HIS3, cdc5-L158G::KanMX6* |
| F3707 | MATa, *SPC42-RFP::KanMX6, SPC72-GFP::HIS3, cdc5-L158G::KanMX6,* |
| F3712 | MATa, *SPC42-RFP::KanMX6, SPC72-GFP::HIS3* |
| F3753 | MATa, *KAR9-sfGFP(cp8)::KanMX4, cdc5-L158G::KanMX6* |
| F3754 | MATa, *SPC110-dsRed::KanMx, KAR9-sfGFP(cp8)::kanMX4* |
| F3755 | MATa, *SPC110-dsRed::KanMx, KAR9-sfGFP(cp8)::kanMX4, cdc5-L158G::KanMX6,* |
| F3766 | MATa, *ura3-1::ADH1-OsTIR1-9Myc(URA3), CDC20-AID::KanMX, cdc5-L158G-yEGFP::HIS3, SPC110-dsRed::KanMX6* |
| F3774 | MATa, *SPC42-RFP::KanMX6, SPC72-GFP::His3, kar9::HIS5* |
| F3780 | MATa, *cdc5-L158G::KanMX6, KAR9-sfGFP(cp8)::KanMX4, SPC42-mCherry::KanMX6* |
| F3836 | MATa, *SPC110-dsRed::KanMx, KAR9-sfGFP(cp8)::KanMX4, spc72::NatMX4* |
| F3897 | MATa, *SPC72-13Myc::His3MX6, cdc5-L158G::KanMX6* |
| F3924 | MATa, *KAR9-13Myc::HIS3MX6* |
| F3964 | MATa, *KAR9-13Myc::His3MX6, cdc5-L158G::KanMX6* |
| F4140 | MATa, *tab6-1-yEGFP::KanMX6, cdc5-L158G::KanMX6, SPC110-dsRed::KanMx* |
| F4439 | MATa, *SPC72-GFP::HIS3, cdc5-2::URA3* |
| F4441 | MATa, *SPC110-dsRed::KanMX6, cdc5-2::URA3* |
| F4498 | MATa, *KAR9-sfGFP(cp8)::KanMX4, cdc5-2::URA3* |
| F4585 | MATa, *TUB4-mScarlet::KanMX6* |
| F4606 | MATa, *SPC72-AA-GFP::HIS3* |
| F4608 | MATa, *SPC72-AA-GFP::HIS3, SPC110-dsRed::KanMx* |
| F4619 | MATa, *cdc5-L158G-yEGFP::HIS3, TUB4-mScarlet::KanMX6* |
| F4628 | MATa, *KAR9-mCherry::NatMX6, SPC72-GFP::HIS3* |
| F4632 | MATa, *KAR9-mCherry::NatMX6, SPC72-AA-GFP::HIS3, cdc5-L158G::KanMX6* |
| F4657 | MATa, *KAR9-13Myc::HIS3MX6, cdc5-2::URA3* |
| F4684 | MATa, *KAR9-mCherry::NatMX6, SPC72-GFP::HIS3, cdc5-L158G::KanMX6* |
| F4696 | MATa, *SPC110-dsRed::KanMX6, cdc5-16::HIS3* |
| F4698 | MATa, *KAR9-sfGFP(cp8)::KanMX4, cdc5-16::HIS3* |
| F4699 | MATa, *SPC72-GFP::KanMX6, cdc5-16::HIS3* |
| F4700 | MATa, *cdc5-16::HIS3, TUB4-mScarlet::KanMX6* |
| F4702 | MATa, *SPC110-dsRed::KanMX6, cdc5-77::HIS3* |
| F4704 | MATa, *KAR9-sfGFP(cp8)::KanMX4, cdc5-77::HIS3* |
| F4705 | MATa, *SPC72-GFP::KanMX6, cdc5-77::HIS3* |
| F4717 | MATa, *KAR9-13Myc::HIS3MX6, cdc5-16::HIS3* |
| F4719 | MATa, *KAR9-13Myc::HIS3MX6, cdc5-77::HIS3* |
| F4734 | MATa, *tab6-1-13Myc::TRP1, cdc5-L158G::KanMX6, SPC110-dsRed::KanMX*, *SPC72-GFP::HIS3* |
| F4754 | MATa, *tab6-1-13Myc::TRP1, cdc5-L158G::KanMX6, SPC110-dsRed::KanMX*, *KAR9-yEGFP::CaURA3* |
| F4756 | MATa, *cdc14-1*, *ura3-1::ADH1-OsTIR1-9Myc(URA3), CDC20-AID::KanMX, cdc5-L158G-yEGFP::HIS3, SPC72-mCherry::NatMX6* |
| F4758 | MATa, *cdc5-2, TUB4-mScarlet::KanMX6* |
| F4820 | MATa, *KAR9-sfGFP(cp8)::KanMX4, ura3::pRS306-mCherry-TUB1::URA3, cdc5-L158G::KanMX6* |
| F4823 | MATa, *KAR9-13Myc::HIS3MX6, TUB4-mScarlet::KanMX6* |
| F4824 | MATa, *KAR9-13Myc::HIS3MX6, spc72::NatMX4* |
| F4825 | MATa, *KAR9-13Myc::HIS3MX6, TUB4-mScarlet::KanMX6*, *SPC72-GFP::HIS3* |
| F4827 | MATa*, cdc5-L158G::KanMX6*, *KAR9-13Myc::HIS3MX6, TUB4-mScarlet::KanMX6*, *SPC72-GFP::HIS3* |
| F4829 | MATa, *KAR9-13Myc::HIS3MX6, TUB4-mScarlet::KanMX6*, *SPC72-AA-GFP::HIS3* |
| F4836 | MATa, *SPC72-GFP::HIS3, SPC110-dsRed::KanMx6, cdc5-L158G::TRP1* |
| F4840 | MATa, *SPC72-AA-GFP::HIS3, SPC110-dsRed::KanMx6, cdc5-L158G:: TRP1* |
| F4842 | MATa*, cdc5-L158G::KanMX6*, *KAR9-13Myc::HIS3MX6, TUB4-mScarlet::KanMX6* |
