# Supplementary material for: Polo-like kinase acts as a molecular timer that safeguards the asymmetric fate of spindle microtubule-organizing centers
Source: eLife. 2020 Nov 2;9:e61488. doi: 10.7554/eLife.61488 (PMC7669271; doi:10.7554/eLife.61488)
Supplement: Supplementary file 2. [file elife-61488-supp2.docx]

**Table S2: Antibodies for immunofluorescence and Western blot**

| Protein | Primary antibody | 1^st^ ab dilution | Secondary  antibody | 2^nd^ ab dilution | Technique |
| --- | --- | --- | --- | --- | --- |
| Tubulin | Anti-tubulin (YOL1/34) monoclonal  (RRID:AB_305329;  Abcam) | 1:250 | Anti-rat FITC  (RRID:AB_2340652;  Jackson  ImmunoResearch) | 1:250 | Immuno-fluorescence |
| Kar9-13Myc | Monoclonal  anti-Myc 9E10  (RRID:AB_291327;  Covance) | 1:5000 | Anti-mouse  HRP-linked  (RRID:AB_772210;GE Healthcare) | 1:10000 | Western  blot |
| Tub4-mScarlet | Monoclonal  anti-RFP 6G6 (RRID:AB_2631395;  Chromotek) | 1:3000 | Anti-mouse  HRP-linked  (RRID:AB_772210;GE Healthcare) | 1:5000 | Western  blot |
| Pgk1 | Monoclonal  anti-Pgk1  (RRID:AB_2532235;  Invitrogen) | 1:20000 | Anti-mouse  HRP-linked  (RRID:AB_772210;GE Healthcare) | 1:10000 | Western  blot |
| Spc72-13Myc | Monoclonal  anti-Myc 9E10  (RRID:AB_291327; Covance) | 1:5000 | Anti-mouse  HRP-linked  (RRID:AB_772210;GE Healthcare) | 1:10000 | Western  blot |
| Spc72-GFP | Monoclonal  anti-GFP Living Colors^®^  (RRID:AB_10013427;Clontech) | 1:1000 | Anti-mouse  HRP-linked  (RRID:AB_772210;GE Healthcare) | 1:3000 | Western  blot |
